# *Mycobacterium marinum mmar_2318* and *mmar_2319* are Responsible for Lipooligosaccharide Biosynthesis and Virulence Toward *Dictyostelium*

**DOI:** 10.3389/fmicb.2015.01458

**Published:** 2016-01-07

**Authors:** Yi-Yin Chen, Feng-Ling Yang, Shih-Hsiung Wu, Tzu-Lung Lin, Jin-Town Wang

**Affiliations:** ^1^Department of Microbiology, National Taiwan University College of MedicineTaipei, Taiwan; ^2^Institute of Biological Chemistry, Academia SinicaTaipei, Taiwan; ^3^Department of Internal Medicine, National Taiwan University HospitalTaipei, Taiwan

**Keywords:** *M. marinum*, lipooligosaccharide, virulence, macrophage, *Dictyostelium*

## Abstract

Resistance to phagocyte killing is an important virulence factor in mycobacteria. *Dictyostelium* has been used to study the interaction between phagocytes and bacteria, given its similarity to the mammalian macrophage. Here, we investigated the genes responsible for virulence to *Dictyostelium* by screening 1728 transposon mutants of the *Mycobacterium marinum* NTUH-M6094 strain. A total of 30 mutants that permissive for *Dictyostelium* growth were identified. These mutants revealed interruptions in 20 distinct loci. Of the 20 loci, six genes (*losA*, *mmar_2318*, *mmar_2319*, *wecE*, *mmar_2323* and *mmar_2353*) were located in the lipooligosaccharide (LOS) synthesis cluster. LOS are antigenic glycolipids and the core LOS structure from LOS-I to LOS-IV have been reported to exist in *M. marinum*. Two-dimensional thin-layer chromatography (2D-TLC) glycolipid profiles revealed that deletion of *mmar_2318* or *mmar_2319* resulted in the accumulation of LOS-III and deficiency of LOS-IV. Deletion and complementation of *mmar_2318* or *mmar_2319* confirmed that these genes both contributed to virulence toward *Dictyostelium* but not entry and replication inside *Dictyostelium*. Co-incubation with a murine macrophage cell line J774a.1 or PMA-induced human monocytic cell line THP-1 demonstrated that *mmar_2318* or *mmar_2319* deletion mutant could grow in macrophages, and their initial entry rate was not affected in J774a.1 but significantly increased in THP-1. In conclusion, although *mmar_2319* has been reported to involve LOS biosynthesis in a previous study, we identified a new gene, *mmar_2318* that is also involved in the biosynthesis of LOS. Deletion of *mmar_2318* or *mmar_2319* both exhibits reduction of virulence toward *Dictyostelium* and increased entry into THP-1 cells.

## Introduction

*Mycobacterium marinum* can cause a systemic tuberculosis-like infection in fish and other ectotherms, a process that involves persistent growth within macrophages (Mehta et al., 2006; Tarigo et al., 2006; Adams et al., 2011; Dong et al., 2012; Hodgkinson et al., 2012; Yang et al., 2012). In humans, this pathogen typically causes only a localized granulomatous infection on cooler surfaces with rare dissemination (Davis and Ramakrishnan, 2009). Macrophages are a first line of defense against bacteria and play a key role in the host’s innate immune response to bacterial infection. In addition, bacteria that have developed resistance to phagocytosis or intracellular killing should be more virulent and more likely to succeed at establishing infection. Mycobacteria that successfully infect macrophages survive and replicate in the phagosome by arresting phagosome maturation and acidification (Vergne et al., 2004; Wong et al., 2011) and damaging the phagosomal membrane to cause macrophage necrosis (Skeiky and Sadoff, 2006; Behar et al., 2010).

The mycobacterial possess a unique lipid-rich cell wall that is important in directing host-pathogen interactions and confers resistance to many therapeutic agents (Jarlier and Nikaido, 1990; Daffe and Draper, 1998). During the infection process, free cell wall lipids/glycolipids are contributing to modulation of the host immune system and condition the outcome of the infection (Karakousis et al., 2004; Neyrolles and Guilhot, 2011). Lipooligosaccharides (LOS) are cell surface glycolipids, and have been reported to exist in more than 10 mycobacterial species, including the *M. canettii*, *M. marinum*, *M. kansasii*, and *M. gastri* (Hunter et al., 1983, 1984; McNeil et al., 1989; Daffe, 1991; Gilleron et al., 1993; Burguiere et al., 2005). All LOS are antigenic compounds containing a α, α’-trehalose unit, the length and composition of LOS are highly variable between different species by different species-specific glycan sequence manner. In *M. marinum*, produces under laboratory conditions, four major LOS structures of increasing size, named LOS-I to LOS-IV, has been previously identified (Burguiere et al., 2005). Loss of LOS results in a rough bacterial colony morphology (Ren et al., 2007; Sarkar et al., 2011), hyper-virulence in zebrafish (van der Woude et al., 2012), reduced biofilm formation, sliding motility, and affect entry rate into macrophages (Ren et al., 2007; Alibaud et al., 2014), inhibition of tumor necrosis factor alpha (TNF-α) secretion in macrophages (Rombouts et al., 2009); and decreased release of proline-glutamic acid_polymorphic guanine-cytosine-rich sequence (PE_PGRS) proteins from the cell surface (van der Woude et al., 2012).

A well-established model system using *Dictyostelium discoideum* was introduced for studying the interactions between phagocytes and bacteria (Solomon et al., 2003; Harriff and Bermudez, 2009; Alibaud et al., 2011). *Dictyostelium*, a free-living amoeba, serves as a macrophage-like system for studying bacteria-host interactions (Solomon et al., 2003). *Dictyostelium* has also been used to analyze the virulence of different bacterial species, including extracellular or intracellular bacteria, such as *Pseudomonas* (Cosson et al., 2002; Pukatzki et al., 2002), *Yersinia* (Vlahou et al., 2009), *Vibrio* (Pukatzki et al., 2006, 2007), *Legionella* (Hilbi et al., 2007; Jules and Buchrieser, 2007; Li et al., 2009), *Klebsiella* (Pan et al., 2011), and *Mycobacteria* (Pozos and Ramakrishnan, 2004; Hagedorn et al., 2009). Upon infection of *Dictyostelium*, *M. marinum* can survive and replicate within intracellular vacuoles, exhibiting a pattern of growth similar to that observed in cultured mammalian macrophages (Hagedorn and Soldati, 2007). Notably, a previous study demonstrated by using a *Dictyostelium* screening model (≤1000 cells) can identify the virulence determinants in *M. marinum* (Alibaud et al., 2011).

As we report here, we constructed a *M. marinum* mutant library by transposon mutagenesis and used a *Dictyostelium* screening model to identify genetic loci involved in *M. marinum* virulence. We identified a new gene, *mmar_2318*, which participates in LOS synthesis and virulence toward *Dictyostelium*.

## Materials and Methods

### Bacterial Strains, Cells, and Growth Conditions

*Mycobacterium smegmatis* mc^2^155 and *M. marinum* NTUH-M6094 (clinically isolated strain from National Taiwan University Hospital) strains were grown at 37°C and 32°C, respectively, in 7H9 medium supplemented with 10% oleic acid/albumin/dextrose/catalase (OADC) enrichment and 0.05% Tween-80. *M. marinum* is a biosafety level-2 microorganism. The experiments handling the bacteria should follow all appropriate guidelines and regulations. *Escherichia coli* and *Klebsiella aerogenes* were grown in Luria broth. Antibiotics were added at the following concentrations when required: kanamycin at 10 mg/L for *M. marinum* and 50 mg/L for *E. coli*; hygromycin at 50 mg/L for *M. marinum* and 100 mg/L for *E. coli*; and ampicillin at 100 mg/L for *E. coli*. *D. discoideum* AX-2 cells were grown at 20°C in HL5 medium (Pan et al., 2011).

### *Dictyostelium* Growth in a Mycobacteria-Phagocytosis Plaque Assay

**FIGURE 1:**
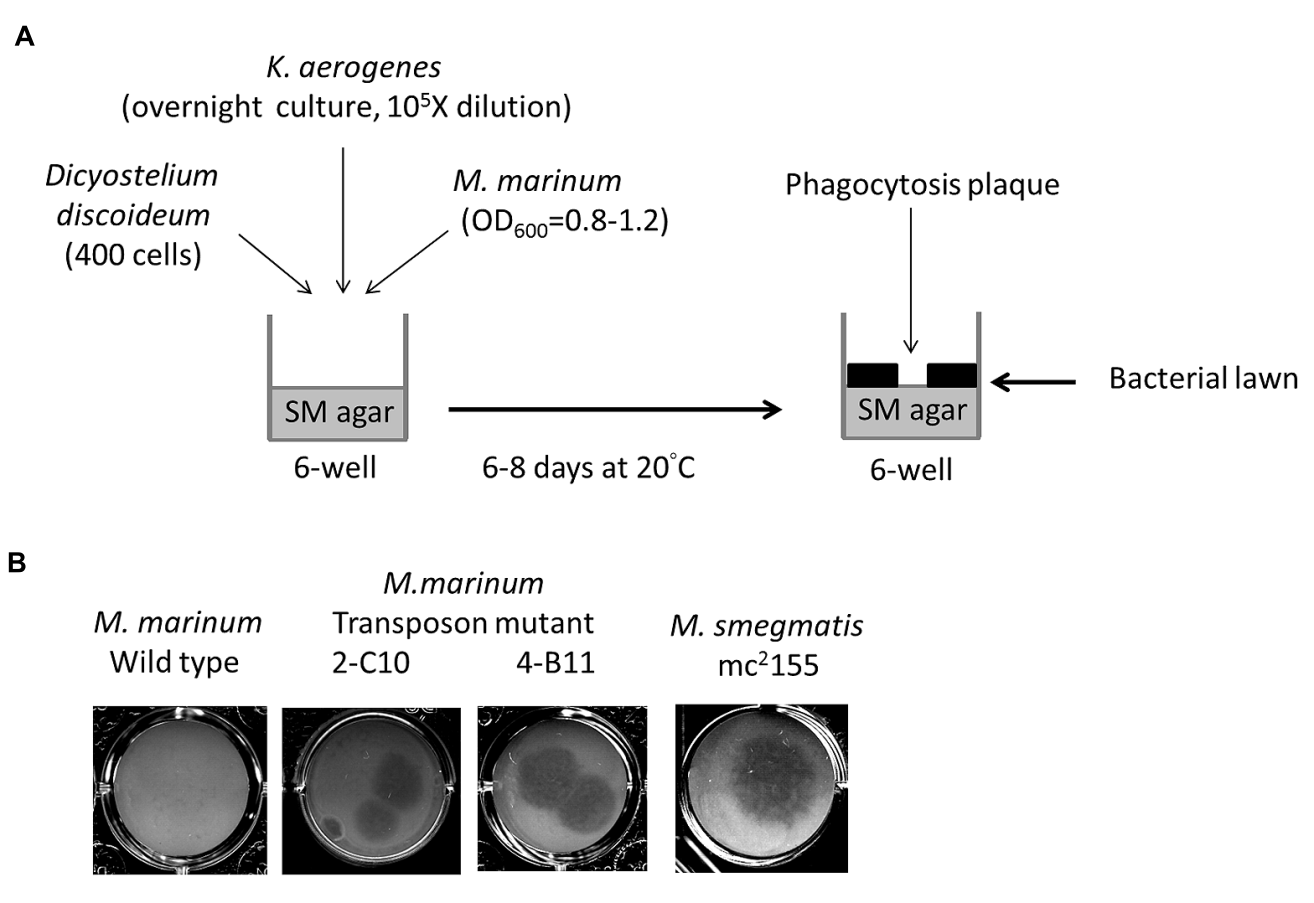
**Identification of *Mycobacterium marinum* genes for virulence using *Dictyostelium.* (A)** Screening method of *M. marinum* NTUH-M6094 mutant library by *Dictyostelium* phagocytosis plaque assay. In six-well tissue culture plates containing SM agar, mid-log phase (*OD*_600_ = 0.8–1.2) cultured *M. marinum* and fresh overnight-cultured *Klebsiella aerogenes* (diluted 10^5^-fold) were mixed in normal saline and then air-dried. Four hundred *Dictyostelium* cells were then added on the top of the bacterial lawn. The plates were incubated at 20°C for 6–8 days until phagocytosis plaques became visible. **(B)** *Dictyostelium* phagocytosis plaques on the *M. marinum* M6094 wild type two transposon mutants and *M. smegmatis*. In presence of wild-type *M. marinum* M6094, *Dictyostelium* failed to form phagocytosis plaques. In contrast, a clear phagocytosis plaque was observed on bacterial lawn with *M. smegmatis* (avirulence mycobacteria). Two mutants 2-C10 and 4-B11 permit the formation of a clear phagocytosis plaque on the bacterial lawn.

The *Dictyostelium* phagocytosis plaque assay was performed as previously described (Bardarov et al., 1997; Alibaud et al., 2011) with some modifications (**Figure 1A**). A 1-mL volume of mid-log phase (*OD*_600_ = 0.8–1.2) *M. marinum* culture was centrifuged and then resuspended with 800 μL of overnight-cultured *K. aerogenes* (as a substrate for *Dictyostelium* when the amoebae were not inhibited by the bacteria) diluted 10^5^-fold in normal saline. The bacterial suspension was plated in six-well (350 μL/well) or 24-well (50 μL) plates containing SM agar (Pan et al., 2011) and then air-dried in a biosafety cabinet for 2 h. *D. discoideum* (400 cells /plate) was then spotted on top of the bacterial lawn. Phagocytosis plaques generated during *D. discoideum* growth became visible after 6–8 days of incubation at 20°C.

### Generation of *M. marinum* Transposon Mutant Library

The TM4-derived conditionally replicating phage phAE94 (a kind gift from Dr. William R. Jacobs, Jr., Howard Hughes Medical Institute, USA; Bardarov et al., 1997) carrying the kanamycin-resistance transposon Tn5367 (Shin et al., 2006) was propagated in *M. smegmatis* mc^2^155 (Bardarov et al., 1997) and used to infect *M. marinum* as described previously (Rybniker et al., 2003).

### Identification of Transposon Mutants by Semi-Random Polymerase Chain Reaction

The insertion site of Tn5367 was determined by semi-random PCR and DNA sequencing as previously described (Chun et al., 1997; Choi et al., 2001; Shin et al., 2006); the primers are listed in **Table 1**.

**Table 1:** Primers and plasmids used in this work.

Primer name	sequence	Purpose	Reference
TnF	TGCAGCAACGCCAGGTCCACACT	Semi-random PCR	
TnR	CAGAAAGTCGTCAGGTCAGC	Semi-random PCR	
HOPS1	GGCGTAGGAACCTCCATCATC	Semi-random PCR	Bardarov et al., 1997
HOPS2	CTTGCTCTTCCGCTTCTTCTCC	Semi-random PCR	Bardarov et al., 1997
semi-rand_2-1	GGCCACGCGTCGACTAGTACNNNNNNNNNNGCAGC	Semi-random PCR	Choi et al., 2001
semi-rand_4	GGCCACGCGTCGACTAGTAC	Semi-random PCR	Choi et al., 2001
2318-Ff-F	ATGAGCATCGCGATGCCCGC	*Δ2318*	
2318-Rf-R-PacI	TTAATTAATCACCGCCTACCTCTTGGCTC	*Δ2318*	
2318-Rf-F	CCTGAATGAGCATTGCCTGTGATGGATGGC	*Δ2318*	
2318-Ff-R	GCCATCCATCACAGGCAATGCTCATTCAGG	*Δ2318*	
2318-promoter-F	GTGCGCTACAAGTTCTAAACC	*Δ2318*::*2318*	
2318-R	CTAATCATCCAGAACTGCTA	*Δ2318*::*2318*	
2319-Rf-R-PacI	TTAATTAAACGAAGTCATCCTGCCGTC	*Δ2319*	
2319-Rf-F	CTGCGGCGCCCGGATTTCACCGCTCATTCA	*Δ2319*	
2319-Ff-R	TGAATGAGCGGTGAAATCCGGGCGCCGCAG	*Δ2319*	
2319-Ff-F	AGGCGTTAGCTACGTGTCGTC	*Δ2319*	
hsp60-F	GGTGACCACAACGACGCGCCC	*Δ2319*::*2319*	
hsp60-R-2319	GAGAGGAGTCTGTCACATGTATATCTCCTTCTTAATTAACTCACCGGT	*Δ2319*::*2319*	
2319-F-hsp60	AGAAGGAGATATACATGTGACAGACTCCTCTCCTCCC	*Δ2319*::*2319*	
2319-R	GTGACAGACTCCTCTCCTC	*Δ2319*::*2319*	

**Plasmid**	**Features**		**Reference**

pMN437	pMN016 derivative, p_smyc_- *gfp*_m_^2+^; pAL5000 origin; Hyg^r^,		Steinhauer et al., 2010
pMN402	Hyg^r^; replicating mycobacterial plasmid with *gfp* under the control of the BCG *hsp60* promoter		Scholz et al., 2000
pGOAL19	Hyg^r^; P_Ag85_-*lacZ* P_hsp60_-*sacB* PacI cassette vector, *amp*		Parish and Stoker, 2000

### Construction of Deletion Mutant

The gene-deleted fragments (Δ*2318* and Δ*2319*) were generated by using the primer pairs listed in **Table 1** and then cloned into a pGEM^®^-T easy (Promega) plasmid. The Hyg^r^-*lacZ*-*sacB* cassette of the pGOAL19 plasmid (Addgene Plasmid #20190; Parish and Stoker, 2000) was digested with PacI and cloned into the PacI site of the resulted plasmid. The vector for gene deletion was transformed into the *M. marinum* NTUH-M6094 strain according to the established procedures (Larsen et al., 2007), and the *M. marinum* deletion mutant was selected after two rounds of homologous recombination, as previously described (Parish and Stoker, 2000).

### Construction of Complementation Strain

*Mycobacterium marinum mmar_2318* (909 bp) and its predicted promoter region (260 bp upstream) were PCR-amplified from genomic DNA using the primer pair 2318-promoter-F/2318-R; *mmar_2319* (1638 bp) and the *M. bovis* BCG *hsp60* promoter region (250 bp) were PCR-amplified from genomic DNA and pMN402 (a kind gift from Dr. Michael Niederweis at the University of Alabama at Birmingham, USA; Scholz et al., 2000) using the primer pairs 2319-F-hsp60/2319-R and hsp60-F/hsp60-R-2319, respectively. The primer pair hsp60-F and 2319-R was then used to PCR amplify the *mmar_2319* gene with the *hsp60* promoter region (1872 bp). Those two PCR products were cloned into a blunted HindIII-site of pMN437 (a kind gift from Dr. Michael Niederweis at the University of Alabama at Birmingham, USA; Steinhauer et al., 2010) to create pMm*2318*::pMN437 and pMm*2319*::pMN437. The complementation strain (Δ*2318*::*2318* and Δ*2319*::*2319*) was created by transforming the pMm*2318*::pMN437 or pMm*2319*::pMN437 plasmid into the Δ*2318* or Δ*2319* strain.

### *Mycobacterium marinum* Lipid Extraction and Analysis

*Mycobacterium marinum* polar and apolar lipids were extracted from fresh-cultured *M. marinum* grown on 7H9 agar plates according to established procedures (Ren et al., 2007). The lipid extract was examined by two-dimensional thin layer chromatography (2D-TLC) (Burguiere et al., 2005). Lipids were visualized by spraying the plates with ceric ammonium molybdate (CAM; 24 g (NH_4_)_6_Mo_7_O_24_⋅4H_2_O, 0.5 g ammonium cerium nitrate, 500 mL H_2_O, 28 mL H_2_SO_4_) followed by gentle charring of the plates.

### Infection of *Dictyostelium* by *M. marinum*

Infection of *Dictyostelium* was performed as described previously (Arafah et al., 2013).

### Infection of J774a.1 or THP-1 Cell Line by *M. marinum*

Infection of murine J774a.1 macrophage-like cells as well as human THP-1 monocytic cell line and enumeration of intracellular *M. marinum* CFU was performed as described previously (Ren et al., 2007). Briefly, a single-cell suspension of fresh cultured *M. marinum* (*OD*_600_ = 0.8–1.0) was yield by passage through a 5-μm syringe filter. The day before experiment, cell were seeding into 24 well [J774a.1, 10^5^ cells/well; THP-1, 10^6^ cells/well, and pre-treatment of THP-1 cell with 50 ng/ml phorbol 12-myristate 13-acetate (PMA) for 48 h]. The cells were infected with bacteria at a multiplicity of infection (MOI) of 1 for growth assays or MOI of 10 for entry rate assays. A previous study indicated that the difference of entry rate between wild type and mutant will be more prominent under MOI of 10 (Alexander et al., 2004). The infection was allowed to proceed for 3 h at 32°C in 5% CO_2_. The extracellular bacteria were removed by washing once with culture medium and incubation in fresh culture medium containing gentamicin (200 mg/L, Gibco^®^) for 2 h at 32°C. The cells were washed once and incubated with fresh culture medium containing 20 mg/L gentamicin at 32°C in 5% CO_2_. On different time point, the infected macrophage monolayers were washed once with culture medium and lysed with 1 mL of 0.1% Triton X-100 (Sigma) for 5 min to release the intracellular mycobacteria. The intracellular bacteria were enumerated by plating serial dilutions on 7H11 agar plates.

### Statistical Analysis

Data are presented as means ± standard error of the mean (SEM) form three independent experiments. Statistical significance was assessed by a two-tailed Student’s *t*-test using Prism 5 (GraphPad Prism^®^) software. *P*-values of <0.05 were considered significant.

## Results

### Screening Mutants Permissive for *Dictyostelium* Growth

We constructed a transposon mutant library of the *M. marinum* strain NTUH-M6094. A total of 1728 mutants were collected. A *Dictyostelium* phagocytosis plaque assay (**Figure 1A**) was used to investigate virulence genes in the M6094 mutant library (Alibaud et al., 2011). As shown in **Figure 1B**, wild-type M6094 did not allow *Dictyostelium* (400 cells) to form a phagocytotic plaque on a bacterial lawn. In contrast, a plaque was observed on a lawn containing *M. smegmatis* mc^2^155 (avirulent mycobacteria). Screening of the entire M6094 mutant library resulted in the identification of 30 transposon mutants that were permissive for *Dictyostelium* growth; examples of the sensitive isolates (two transposon mutants, 2-C10 and 4-B11) are presented in **Figure 1B**. This phenotype implied that the genes disrupted by the transposon are potentially involved in virulence. The genes interrupted by transposons were determined by semi-random PCR (Chun et al., 1997; Shin et al., 2006) and DNA sequencing (**Table 2**). The results indicated that 20 genes were disrupted by the transposon, and the locations of the transposon insertion in the 30 mutants were unique.

**Table 2:** Transposon mutants permissive for *Dictyostelium* growth.

Mutant No.	Genes inserted by transposon	Putative function	Homologs in *M. tuberculosis* H37Rv
8-H11	*mmar_0328*	Secreted antigen 85-C	FpbC
12-E12	*mmar_0838*	Hypothetical protein	
14-C12	*mmar_0932*	PPE family protein	PPE24
14-F4	*mmar_1514*	PPE family protein, PPE51_1	PPE51
12-B3, 12-E1	Upstream of *mmar_1594* and *mmar_1595*	*mmar_1594*: PE_PGRS family protein *mmar_1595*:O-methyltransferase	PE_PGRS55 Rv3767c
15-B4	*mmar_1639*	PPE family protein	PPE8
12-C12	*mmar_1887*	Conserved transmembrane transport protein	
4-B11, 4-C3, 16-G9	*mmar_2313*	*losA*, glycosyltransferase	Rv1500
14-D5, 2-A3, 4-E9, 15-D8	*mmar_2318*	Conserved hypothetical protein	Rv1502
16-F5, 11-G3, 2-E6	*mmar_2319*	Conserved hypothetical transmembrane protein	
2-C10, 2-G4	*mmar_2320*	*wecE*, pyridoxal phosphate-dependent enzyme	
10-A11	*mmar_2323*	Conserved hypothetical transmembrane protein	
13-B8	*mmar_2353*	UDP-glycosyltransferase	Rv1524
12-E11	Upstream of *mmar_2684*	PPE family protein	PPE32
18-G4	*mmar_3183*	Hypothetical alanine rich protein	
18-D7	Upstream of *mmar_3375*	Conserved hypothetical protein	
17-A5, 18-H5, 12-A1	*mmar_4263*	Conserved hypothetical protein	
13-G8	*mmar_4621*	PPE family protein	PPE8
5-H1	*mmar_4630*	Membrane-bound C-5 sterol desaturase	Erg3

### Defective LOS Biosynthesis in the Deletion Mutant

**FIGURE 2:**
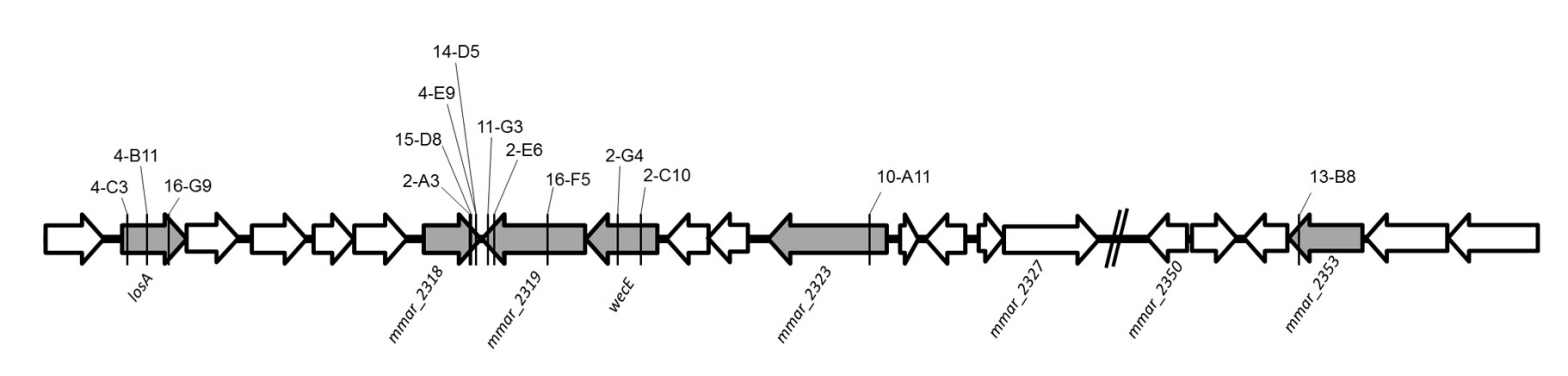
**The transposon insertion sites of mutants within lipooligosaccharide (LOS) synthesis locus**. The six genes with gray color (*losA*, *mmar_2318*, *mmar_2319*, *wecE*, *mmar_2323* and *mmar_2353*) within LOS synthesis locus had transposon insertions in different sites. The solid line indicates the transposon insertion.

**FIGURE 3:**
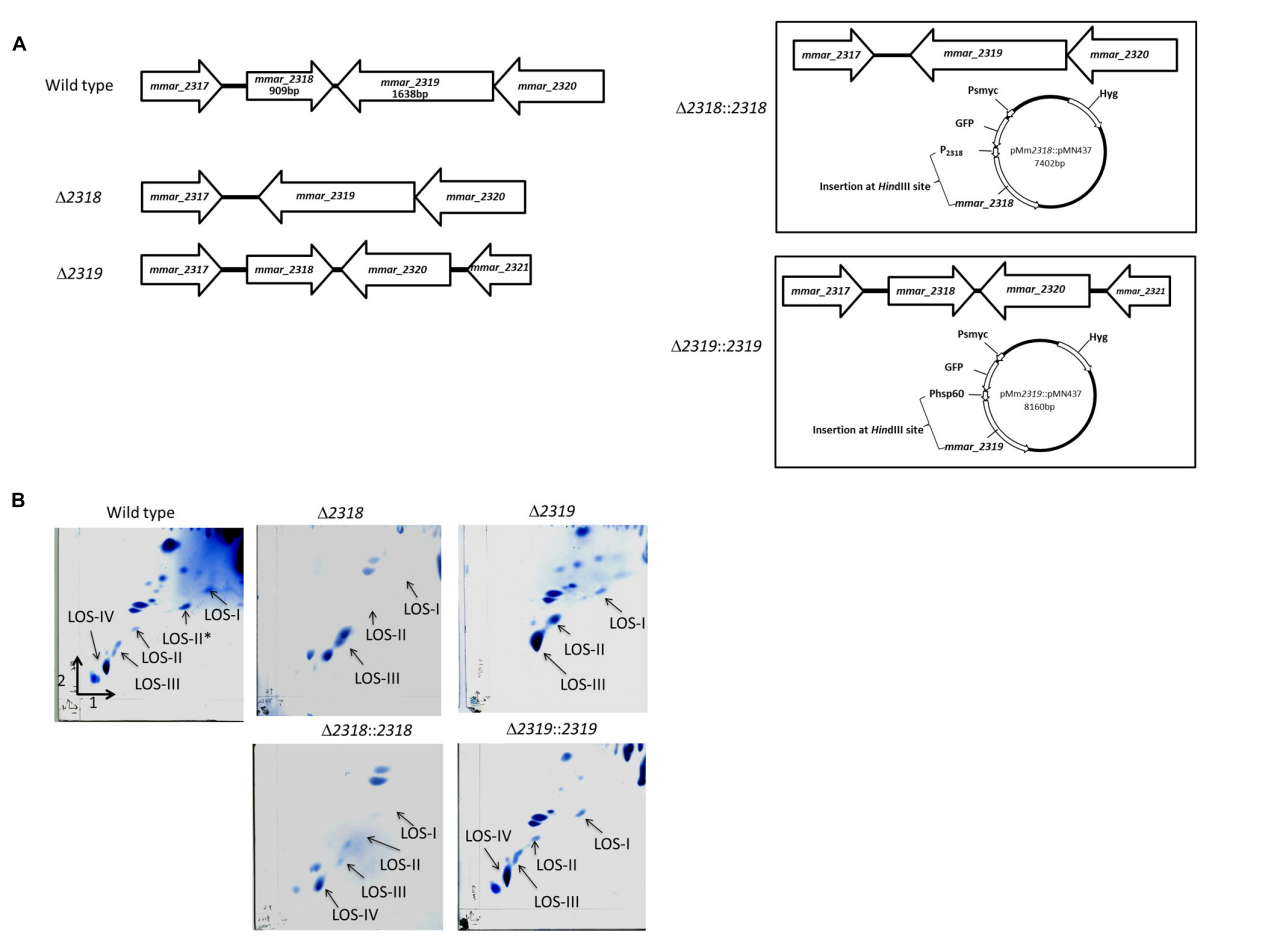
**The 2D-TLC profile of the polar lipid of *M. marinum* wild-type, deletion mutants and complementation strains. (A)** The gene alignment of *mmar_2318* and *mmar_2319* deletion mutants and complementation strains. The Δ*2318* and Δ*2319* deletion mutants were created by unmarked deletion of *mmar_2318* and *mmar_2319*, respectively. pMm*2318*::pMN437 and pMm*2319*::pMN437 plasmids were used to create the epichromosomal complementation strains Δ*2318*::*2318* and Δ*2319*::*2319*, respectively. **(B)** The 2D-TLC profile of the polar lipid of *M. marinum*. Using 2D-TLC, extracted *M. marinum* polar lipids were separated by chloroform/methanol/water (60:30:6, v/v/v) in the first direction and by chloroform/acetic acid/methanol/water (40:25:3:6, v/v/v/v) in the second direction. The plates were charred with ceric ammonium molybdate. Accumulation of LOS-III and deficiency of LOS-IV were observed in the Δ*2318* and Δ*2319* mutants. The 2D-TLC lipid composition profiles of the Δ*2318*::*2318* and Δ*2319*::*2319* complementation strains were restored to that of the wild-type strain.

Among the 30 attenuated mutants, the transposons of three mutants (4-B11, 4-C3, 16-G9) were inserted into different sites of *losA*, four mutants (2-A3, 4-E9, 14-D5, 15-D8) into *mmar_2318*, three mutants (2-E6, 11-G3, 16-F5) into *mmar_2319*, two mutants (2-C10, 2-G4) into *wecE*, one mutant into *mmar_2323* (10-A11) and one mutant into *mmar_2353* (13-B8) (**Figure 2**). As presented in **Table 2** and **Figure 2**, these six genes (*losA*, *mmar_2318*, *mmar_2319*, *wecE*, *mmar_2323*, and *mmar_2353*) are located within a putative LOS biosynthetic gene cluster (*mmar_2307*∼*mmar_2405*) (Ren et al., 2007; van der Woude et al., 2012). Previous studies reported that *losA*, *mmar_2319*, *wecE* and *mmar_2353* are involved in LOS biosynthesis (Domenech et al., 2005; Brodin et al., 2010). Here, two genes, *mmar_2318* and *mmar_2319*, which were identified in several attenuated mutants in this study were chosen for further studies. Deletion mutants (Δ*2318* and Δ*2319*) and episomal complementation strains (Δ*2318*::*2318* and Δ*2319*::*2319*) were generated accordingly (**Figure 3A**). The surface polar lipid profiles of wild-type, deletion mutants (Δ*2318* and Δ*2319*), and complementation strains (Δ*2318*::*2318* and Δ*2319*::*2319*) were examined by 2D-TLC (**Figure 3B**). We referred to several previous studies to predict the pattern of lipid migration on TLC plate (Alexander et al., 2004; Burguiere et al., 2005; van der Woude et al., 2012; Alibaud et al., 2014). The 2D-TLC spots of *losA*::Tn and *wecE*::Tn (Supplementary Figure S1) mutants which have been reported to have defective LOS biosynthesis were also served as controls. The result indicates that Δ*2318* and Δ*2319* mutants exhibited accumulation of LOS-III and deficiency of LOS-IV. The 2D-TLC profile of the complementation strains (Δ*2318*::*2318* and Δ*2319*::*2319*) were restored to that of wild type. Although LOS-IV deficiency of a *mmar_2319* transposon mutant has been demonstrated in previous studies (van der Woude et al., 2012; Alibaud et al., 2014), the role of this gene in LOS synthesis was confirmed by deletion and complementation in this study. These data suggested that these two genes were responsible for LOS synthesis.

### Phenotypic Confirmation Using Deletion and Complementation Strains

**FIGURE 4:**
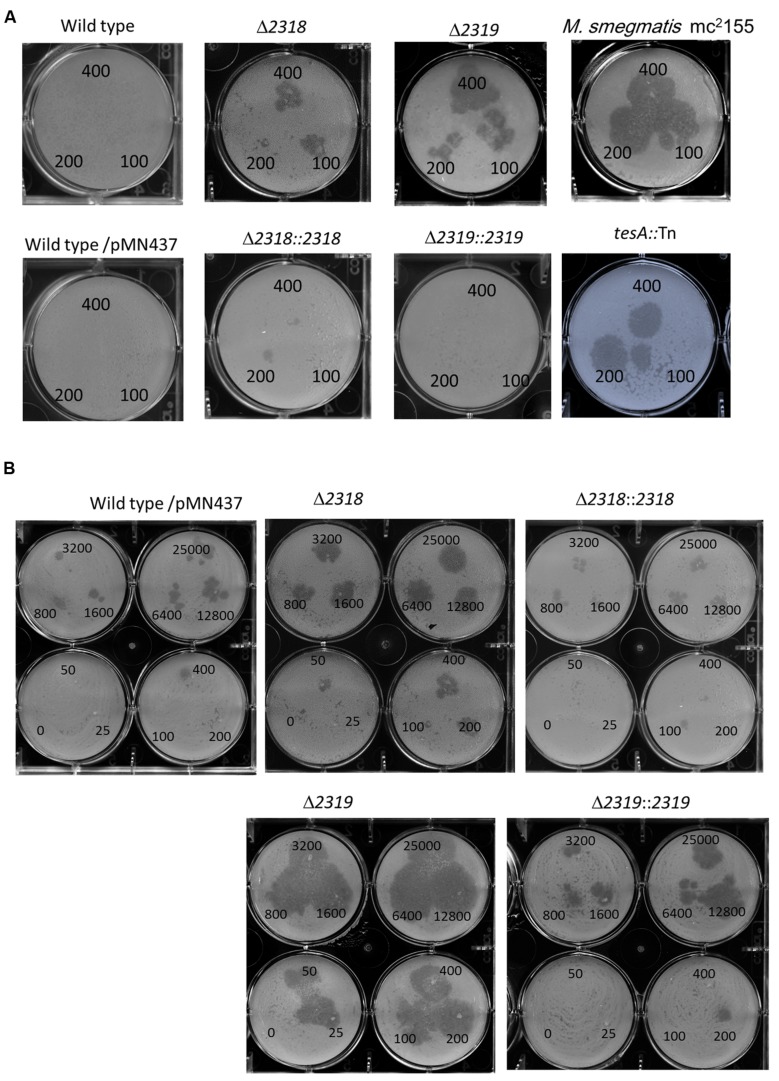
**Phenotypic confirmation and quantitative analysis of deletion mutants and complementation strains. (A)** The *Dictyostelium* phagocytosis plaques on bacterial lawn with the wild-type, deletion, and complementation strains. A total of 400, 200, or 100 *Dictyostelium* cells could form a clear phagocytosis plaque on a bacterial lawn with Δ*2318* and Δ*2319* mutants but not with the wild-type, Δ*2318*::*2318* and Δ*2319*::*2319* strains. *M. smegmatis* mc^2^155 and *tesA*::Tn mutant (known as an avirulent *M. marinum* mutant) were served as controls. Comparable results were observed in wild type vs. wild type/pMN437, demonstrating that the transfer of pMN437 into *M. marinum* did not affect phagocytic plaque formation. **(B)** Quantitative analysis of virulence of wild-type, deletion, and complementation strains. Different amounts of *Dictyostelium* (0–25000 cells) were used to quantify the virulence of the *M. marinum* wild-type strain, the deletion mutants (Δ*2318* and Δ*2319*), and the complementation strains (Δ*2318*::*2318* and Δ*2319*::*2319*). Fifty cells and twenty-five *Dictyostelium* cells could form a clear phagocytic plaque on bacterial lawn with the Δ*2318* and Δ*2319* mutants, respectively, but not with the wild-type, Δ*2318*::*2318* and Δ*2319*::*2319* strains. The virulence phenotype was restored (>400 cells) in the complementation strain.

First, deletion of *mmar_2318* or *mmar_2319* did not significantly affect the growth rate at 32°C and 20°C [Supplementary Figure S2 and (Solomon et al., 2003)], indicating that the attenuation of these two mutants were not due to *in vitro* growth defect. The *mmar_2318* and *mmar_2319* deletion and complementation strains exhibited the expected attenuation and virulence phenotypes in the *Dictyostelium* phagocytotic plaque assay (**Figure 4A**), confirming the results observed with the transposon mutants. Meanwhile, a *tesA* transposon mutant with attenuation phenotype which has been reported previously (Alibaud et al., 2011) was generated and served as a control (**Figure 4A**). Furthermore, we also used different cell numbers of *Dictyostelium* to quantify the virulence of the wild-type, deletion mutant, and complementation strains. In presence of wild-type *M. marinum*, phagocytic plaque formation exhibited a dose-dependent response to the *Dictyostelium* cell number, with plaque formation detected in the presence of >400 amoeba (**Figure 4B**). Deletion of *mmar_2318* or *mmar_2319* attenuated the virulence to *Dictyostelium* (with plaques observed with 50 amoeba cells and as few as 25 amoeba cells, respectively), whereas two complementation strains restored the virulence to *Dictyostelium* (>400 cells) to a level similar to that observed in wild type.

The loss of LOS results in rough bacterial colony morphology (Ren et al., 2007; Sarkar et al., 2011; van der Woude et al., 2012). The Δ*2318* and Δ*2319* mutants showed a rough phenotype and bigger colonies size than wild type (Supplementary Figure S3). Colonies morphology and size of complementation strains (Δ*2318*::*2318* and Δ*2319*::*2319*) were restored as those of wild type (Supplementary Figure S3).

### Deletion of *mmar_2318* or *mmar_2319* did not Affect the Entry and Replication Inside *Dictyostelium*

**FIGURE 5:**
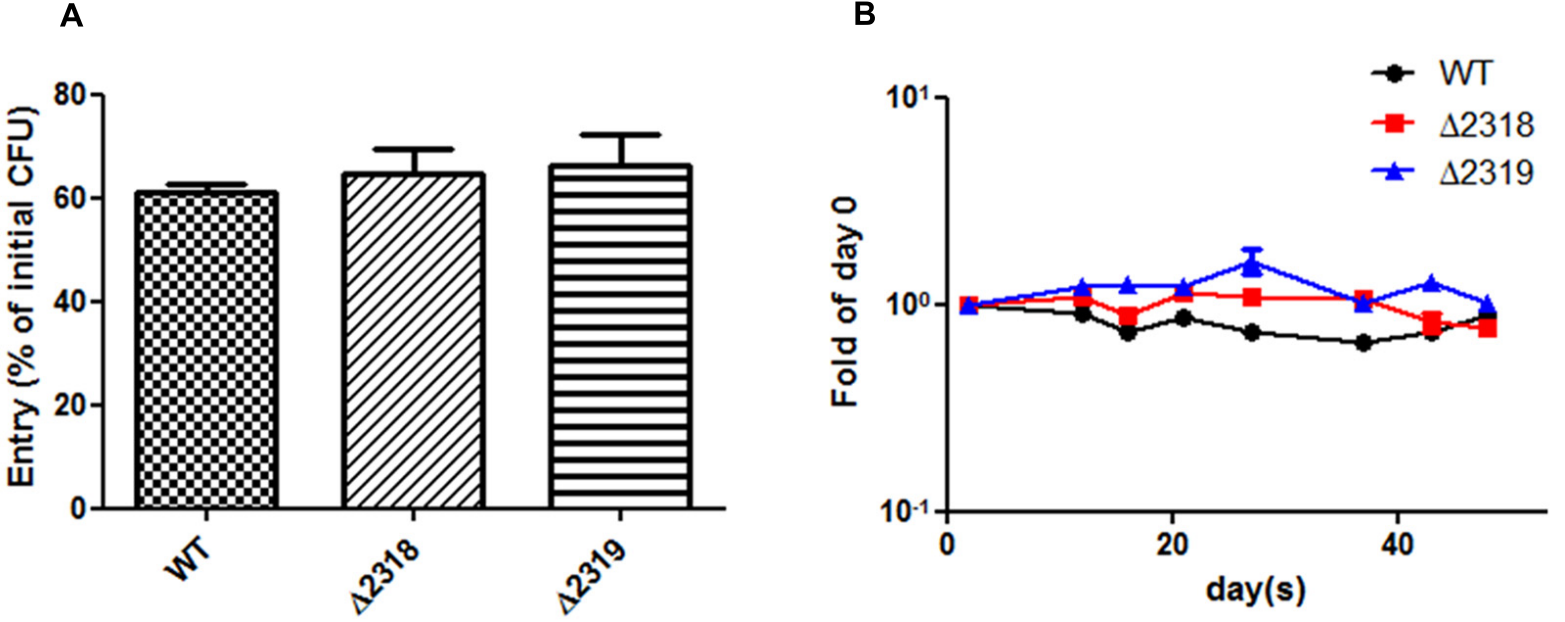
**Entry and growth of *M. marinum* inside *Dictyostelium.* (A)** The entry of *M. marinum* wild-type, Δ*2318* and Δ*2319* into *Dictyostelium*. The bacteria numbers inside *Dictyostelium* cells on 2 h post infection at *MOI* = 10 were determined. Entry was represented as the percentage of entry CFU versus initial CFU of three independent experiments. The two deletion mutants (Δ*2318* and Δ*2319*) had no significantly different entry rate in comparison with wild type. Data from three independent experiments are presented as the mean ± SEM of the % of initial CFU. **(B)** Growth kinetics of the *M. marinum* wild-type, Δ*2318* and Δ*2319* strain inside *Dictyostelium*. The bacteria numbers inside *Dictyostelium* on different hours (2, 12, 16, 21, 26, 37, 43, 48) post infection at *MOI* = 10 were determined. Data from three independent experiments are presented as the mean ± SEM of the fold of initial entering bacteria number.

Deletion and complementation confirmed *mmar_2318* and *mmar_2319* were contributed to virulence toward *Dictyostelium*. We further examined the ability of the Δ*2318* and Δ*2319* strains to enter and replicate inside cells of *Dictyostelium*. The results showed that the number of CFU recovered from *Dictyostelium* was not significantly different between wild type and mutants (Δ*2318* and Δ*2319*) (**Figure 5A**). The growth rate of Δ*2318* or Δ*2319* mutants inside *Dictyostelium* was subsequently monitored, as shown in **Figure 5B**, the growth rate of wild type and mutants were not significantly different, either. These results indicated that deletion of *mmar_2318* or *mmar_2319* did not affect the entry and replication inside *Dictyostelium*.

### Increased Entry to THP-1 Macrophage Cells in the Deletion Mutants

**FIGURE 6:**
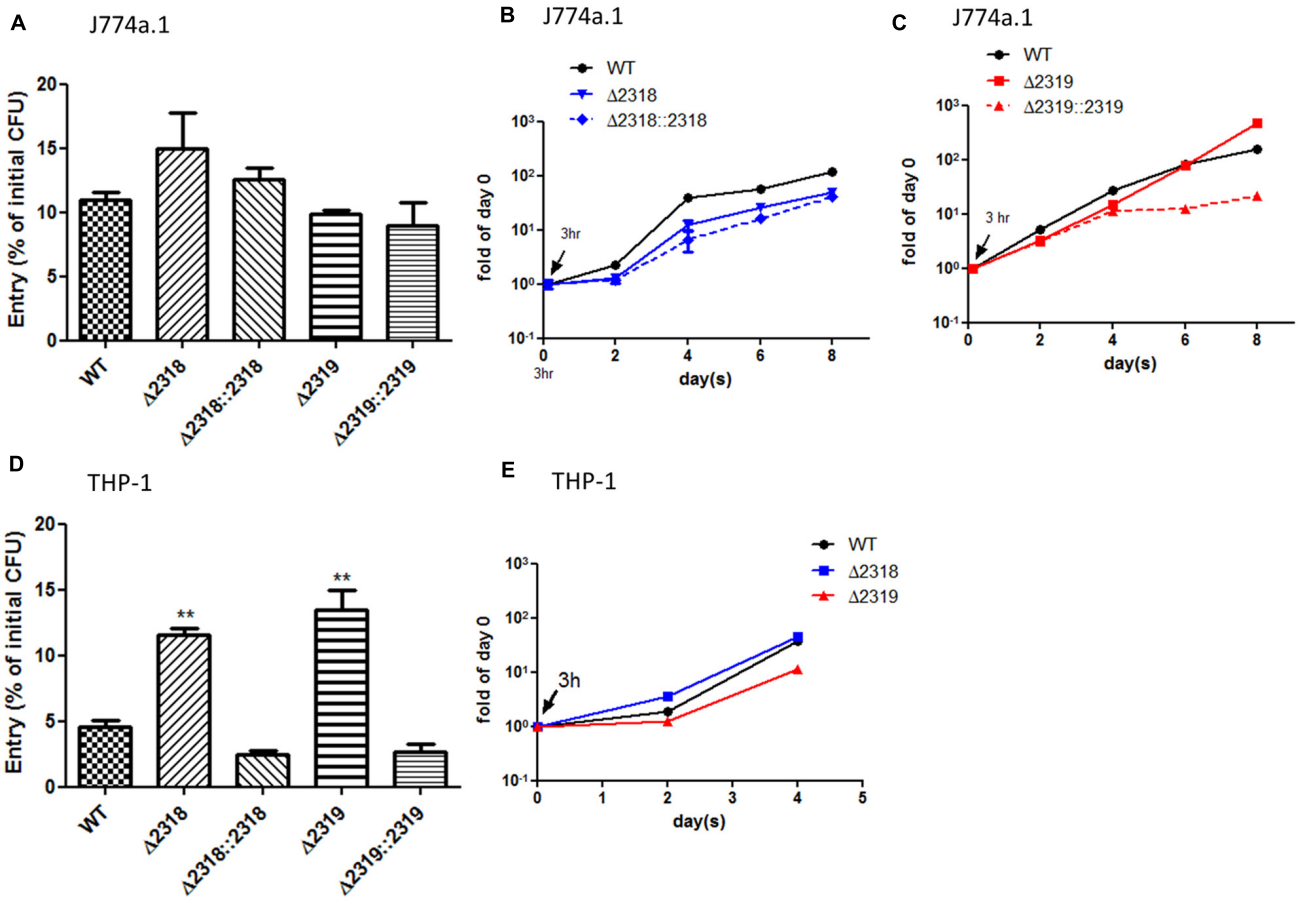
**Entry and growth of *M. marinum* inside macrophages**. The entry of *M. marinum* wild-type, Δ*2318*, Δ*2319*, Δ*2318*::*2318*, and Δ*2319*::*2319* strains into macrophages. The bacteria numbers inside macrophage cells on day 0 (3 h post infection) at *MOI* = 10 were determined. Entry was represented as the percentage of entry CFU vs. initial CFU of three independent experiments. No significant differences were noted among the wild-type strain, the deletion mutants (Δ*2318* and Δ*2319*), and the complementation strains (Δ*2318*::*2318* and Δ*2319*::*2319*) into J774a.1 **(A)** but the recovered bacteria number of two deletion mutants were significantly higher than wild-type strain into THP-1 **(D)**. Data from three independent experiments are presented as the mean ± SEM of the % of initial CFU. ^∗∗^*p* < 0.001. Growth kinetics of the *M. marinum* wild-type, Δ*2318*, Δ*2319*, Δ*2318*::*2318*, and Δ*2319*::*2319* strain inside macrophages. The bacteria numbers inside J774a.1 **(B,C)** or THP-1 **(E)** cells on days 0 (3 h), 2, 4, 6, and 8 days at *MOI* = 1 were determined. Data from three independent experiments are presented as the mean ± SEM of the fold of initial entering bacteria number.

Besides observations in *Dictyostelium*, the ability of the Δ*2318* and Δ*2319* strains to enter and replicate inside cells of macrophage cell line, J774a.1 (murine) and THP-1 (human), were examined. After incubation of bacteria and macrophage cell lines for 3 h at 32°C, the cultures were then treated with gentamicin to remove extracellular bacteria, and intracellular bacteria were quantified by lysing the infected cultures and plating onto 7H11 agar plates. The results indicated that the entry abilities of Δ*2318* and Δ*2319* mutants were not affected in J774a.1 (**Figure 6A**), but the number of CFU recovered from THP-1 cell line infected with the Δ*2318* or Δ*2319* mutants was significantly higher than that infected with the wild-type strain (**Figure 6D**). We also examined the subsequent growth of Δ*2318* and Δ*2319* mutants inside these two macrophage cell lines. The results demonstrated that the deletion mutants both exhibited growth rates similar to wild type and could replicate inside macrophages during the course of infection (**Figures 6B,C,E**). These data suggest that deletion of *mmar_2318* or *mmar_2319* did not affect the ability of the bacteria to replicate within macrophages.

## Discussion

The complete genome of the *M. marinum* M strain is approximately 6.6 Mb in size and is composed of 5568 open reading frames (ORFs) (accession number: CP000854.1). Our mutant library includes a collection of 1728 transposon insertions. The insertion sites of 30 *Dictyostelium*-permissive mutants (in the present work), as well as those of another 17 randomly selected mutants (**Table 3**), were unique, and the transposons of these 47 mutants were collectively located in 36 different loci (**Tables 2** and **3**). These results indicate that the library has good diversity. However, real coverage is difficult to estimate given that insertions close to an operon can cause phenotypic changes due to polar effects on gene expression. Additionally, essential genes (estimated as 5–20% of bacterial genomes) (Gerdes et al., 2003; Salama et al., 2004; Liberati et al., 2006), although less likely to be specific virulence genes, would not be identified by transposon mutagenesis. Therefore, we expect that our mutant library is not saturated with respect to candidate loci, and our screen and library are expected to have missed multiple loci.

**Table 3:** Seventeen randomly selected transposon mutants for diversity check.

Mutant No.	Genes inserted by transposon	Putative function
1	*mmar_1131*	Hypothetical protein
2	*mmar_1485*	Membrane-associated phospholipase C 2 PlcB_2
3	*mmar_3589*	Prophage integrase
4	*mmar_2513*	Hypothetical protein
6-F5	*mmar_4264*	Conserved hypothetical protein
6-F7	*mmar_3382*	Conserved hypothetical membrane protein
6-F10, 10-B11	Not similar with sequences of *M. marinum* M (may be the same gene but different insertion sites)	
6-G2	Not similar with sequences of *M. marinum* M	
6-G3	Not similar with sequences of *M. marinum* M	
9-D1	*mmar_5435*	Conserved hypothetical alanine and glycine rich protein
9-D12	*mmar_2687*	Mg^2+^ transport p-type ATPase C MgtC
9-E2	*mmar_3612*	Metal cation transporter p-type ATPase
9-E5	*mmar_0932*	PPE family protein
9-E6	Not similar with sequences of *M. marinum* M	
10-B9	*mmar_0599*	Conserved hypothetical secreted protein
10-C2	*mmar_3414*	Hypothetical alanine and proline rich protein

Alibaud et al. (2011) first used *Dictyostelium* as a screening model to identify virulent genes within *M. marinum* in 2011. These researchers screened only 275 transposon mutants of the *M. marinum* M strain (Alibaud et al., 2011). Our work adopted the same screening strategy, but we screened a larger number of mutants (1728 isolates, corresponding to our entire mutant library) and used a distinct strain of *M. marinum*. In this study, we used a clinical isolate, the *M. marinum* NTUH-M6094 strain, to construct the mutant library. When assessing the library diversity, we also found that 5/36 (13.8%) of the transposon interrupted loci were not obvious homologs of sequences from the sequenced *M. marinum* M strain (**Table 3**). This result suggests that genetic heterogeneity exists in *M. marinum* isolates from different regions and/or sources.

In this study, we used a *Dictyostelium* phagocytotic plaque model system to screen a *M. marinum* transposon mutant library, identifying 20 genes with roles in virulence. Among these 20 genes, some loci [e.g., *losA, wecE*, transmembrane transporter protein and proline-proline-glutamic acid (PPE) family related genes] were previously reported to be associated with virulence, macrophage resistance, biofilm formation, or LOS synthesis in *Mycobacterium* spp. (Okkels et al., 2003; Burguiere et al., 2005; Domenech et al., 2005; McEvoy et al., 2009; Brodin et al., 2010; Alibaud et al., 2011; Dong et al., 2012; Wang et al., 2013). The results of our screen were therefore consistent with data from other studies. We also found several genes that were not previously identified as virulence genes in the literature, including 12 loci with homologs in *M. tuberculosis* (**Table 2**). Our results implicate these loci in *M. tuberculosis* pathogenesis. However, the actual role of these genes in *M. marinum* and *M. tuberculosis* will require confirmation; characterization of these loci will be reported elsewhere.

In our study, six genes (*losA*, *mmar_2318*, *mmar_2319*, *wecE*, *mmar_2323*, and *mmar_2353*) located within the predicted LOS synthesis locus were identified. The effects on the polar lipid 2D-TLC profile after transposon knockout of *losA*, *mmar_2319*, *wecE*, and *mmar_2353* were previously reported (van der Woude et al., 2012; Alibaud et al., 2014) and confirmed by this study (**Figure 3B** and Supplementary Figure S1). In this study, we focused on the role of *mmar_2318* and *mmar_2319* on LOS synthesis and virulence in *Dictyostelium* and macrophages. Although deletion of *mmar_2318* or *mmar_2319* both revealed deficiency of LOS-IV, prominent phagocytosis plaques and bigger colonies were observed in *Δ2319* mutant compared with *Δ2318* mutant. This may be due to different degrees of LOS-III accumulation or unknown effects other than impairment of LOS after deletion of *mmar_2318* or *mmar_2319*.

Our result confirmed Δ*2318* and Δ*2319* mutants also reduced virulence toward *Dictyostelium.* To dissect the virulence toward amoebae contributed by *mmar_2318* and *mmar_2319*, we also examined the ability of the Δ*2318* and Δ*2319* mutants to enter and replicate inside cells of *Dictyostelium*. As shown in **Figure 5**, no matter entry or replication inside *Dictyostelium*, there were no significant difference between wild-type and two deletion mutants. These results indicated that the reduced virulence toward *Dictyostelium* after deletion of *mmar_2318* or *mmar_2319* was not resulted from affecting the initial entry and survival inside cells. These two genes might be through other mechanisms to inhibit *Dictyostelium* growth.

In 2012, van der Woude et al. (2012) found that a *M. marinum wecE* transposon mutant was hyper-virulent to zebrafish, but our study demonstrated that a *wecE*::Tn mutant was permissive for *Dictyostelium* growth. This difference is potentially attributed to the facts that zebrafish and *Dictyostelium* are different species and zebrafish is a more complex model. These results suggested that the attenuation of mutants identified by using *Dictyostelium* screening should be confirmed in a more complex host. A recent study published by Alibaud et al. (2014) examined the phagocytosis of a *mmar_2319* transposon mutant by murine macrophage J774a.1 cells. This study is consistent with our observation that the phagocytosis rate of the *mmar_2319* deletion mutant was similar to that of the wild-type strain in J774a.1. But in this study, we observed the entry ability of deletion mutants (Δ*2318* or Δ*2319*) was significantly higher than that of wild type into another macrophage cell line, THP-1. This may be due to different host origins that these two cell lines were isolated from. The increased entry into THP-1 cell after deletion of *mmar_2318* and *mmar_2319* might explain the hyper-virulence to zebrafish of *wecE* mutant which also revealed accumulation of LOS-III and deficiency of LOS-IV. However, the virulence of Δ*2318* and Δ*2319* mutants to zebrafish or mammalian hosts requires more investigations.

## Conclusion

We identified a new gene, *mmar_2318*, involved the LOS biosynthesis. *M. marinum mmar_2318* and *mmar_2319* were both responsible for virulence toward *Dictyostelium*; deletion of *mmar_2318* and *mmar_2319* increased entry ability into THP-1 cell but not affected the replication inside *Dictyostelium* and macrophages.

## Conflict of Interest Statement

The authors declare that the research was conducted in the absence of any commercial or financial relationships that could be construed as a potential conflict of interest.
