# Supplementary material for: Mycobacterium marinum mmar_2318 and mmar_2319 are Responsible for Lipooligosaccharide Biosynthesis and Virulence Toward Dictyostelium
Source: Front Microbiol. 2016 Jan 7;6:1458. doi: 10.3389/fmicb.2015.01458 (PMC4703794; doi:10.3389/fmicb.2015.01458)
Supplement: Supplementary file 1 [file Data_Sheet_1.PDF]

Supplementary Material

Mycobacterium marinum mmar_2318 and *mmar_2319* are Responsible for Lipooligosaccharide Biosynthesis and Virulence towards *Dictyostelium*

Heading title: *mmar_2318* and *mmar_2319* in *M. marinum*

Yi-Yin Chen[a], Feng-Ling Yang[b], Shih-Hsiung Wu[b], Tzu-Lung Lin[a]*, Jin-Town Wang[a,c]*

[a]Department of Microbiology, National Taiwan University College of Medicine, Taipei, Taiwan;

[b]Institute of Biological Chemistry, Academia Sinica, Taipei, Taiwan;

[c]Department of Internal Medicine, National Taiwan University Hospital, Taipei, Taiwan

* Correspondence:

Jin-Town Wang, M.D., Ph.D. or Tzu-Lung Lin Ph.D.

T.L.L. and J.T.W. contributed equally to this work.

Department of Microbiology

National Taiwan University College of Medicine

1, Sec 1, Jen-Ai Rd.

Taipei, Taiwan.

wangjt@ntu.edu.tw or f87445101@ntu.edu.tw

Keywords: *M. marinum*; lipooligosaccharide; virulence; macrophage; *Dictyostelium*

Supplementary Figures

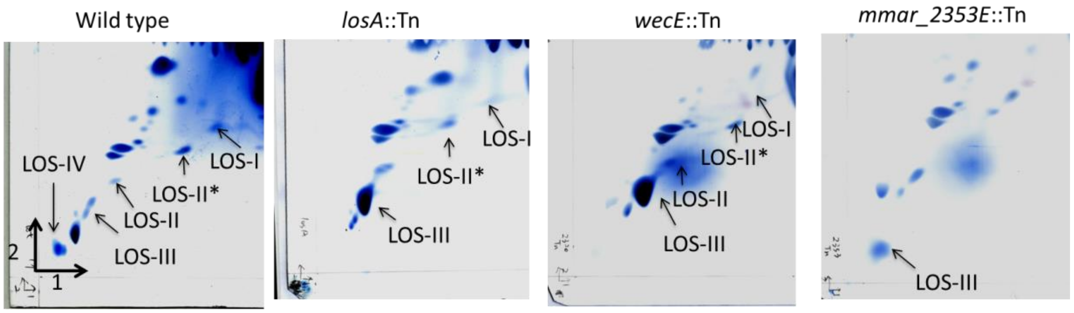

Figure S1. The 2D-TLC profile of the polar lipid of *M. marinum*

Accumulation of LOS-III and deficiency of LOS-IV were observed in *losA*::Tn and *wecW*::Tn mutants; all LOS-deficient was observed in *mmar_2353*::Tn mutants.

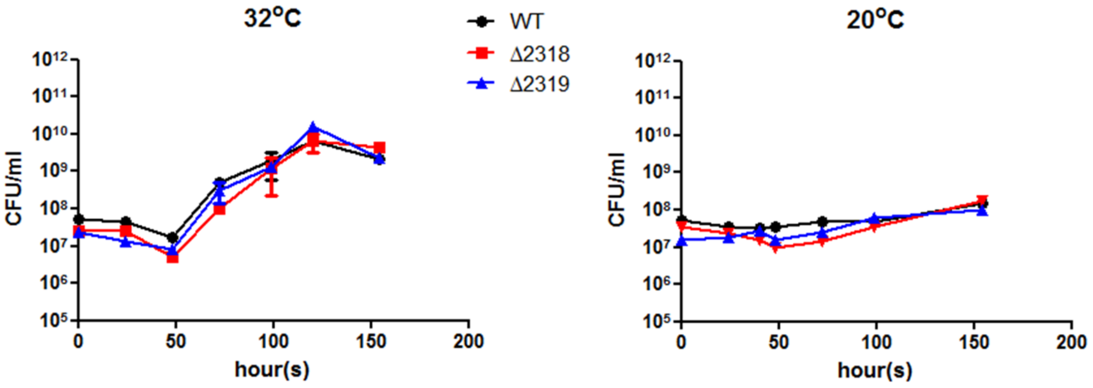

Figure S2. Growth curves of wild-type, *Δ2318* and *Δ2319* mutants at 32°C and 20°C

Growth curves of bacterial cultures in 7H9, 0.05% Tween 80, and 10% OADC enrichment at 32°C and 20°C were represented by mean CFU/ml with standard error of the mean (SEM) of three independent cultures.

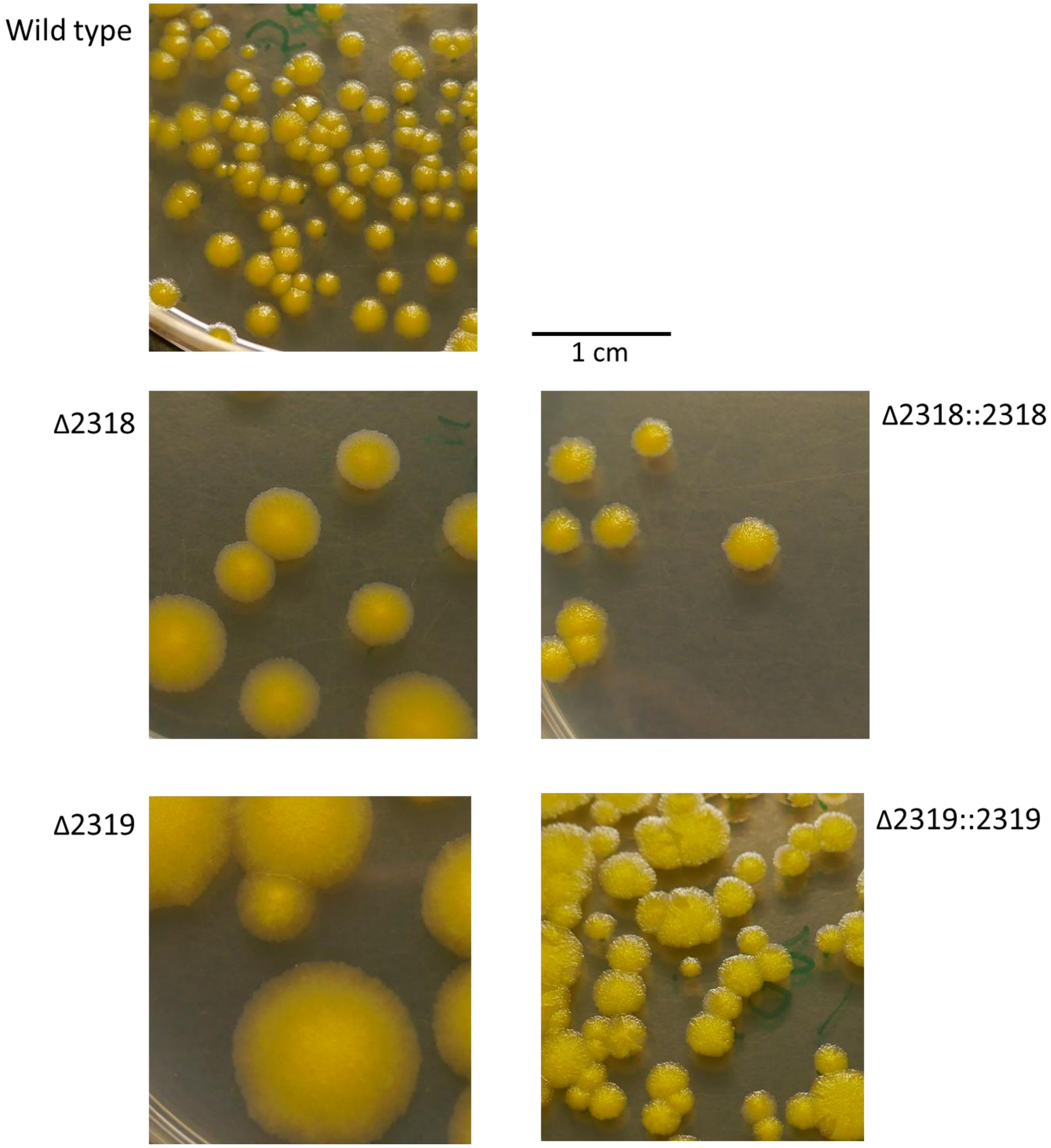

Figure S3. Colony morphology of wild-type, *Δ2318*, *Δ2319* and complementation strains

Δ2318 and *Δ2319* mutants revealed a rough colony morphology and bigger colony size than wild-type. The complementations restored colony morphology and colony size as wild-type did.
